# Antibiotic resistance in the wild: an eco-evolutionary perspective

**DOI:** 10.1098/rstb.2016.0039

**Published:** 2017-01-19

**Authors:** Teppo Hiltunen, Marko Virta, Anna-Liisa Laine

**Affiliations:** 1 Department of Food and Environmental Sciences/Microbiology and Biotechnology, University of Helsinki, PO Box 65, 00014 Helsinki, Finland; 2 Department of Biosciences, Metapopulation Research Centre, University of Helsinki, PO Box 65, 00014 Helsinki, Finland

**Keywords:** antimicrobial resistance, horizontal gene transfer, eco-evolutionary dynamics, evolution, microbial community dynamics, sub-inhibitory antibiotics

## Abstract

The legacy of the use and misuse of antibiotics in recent decades has left us with a global public health crisis: antibiotic-resistant bacteria are on the rise, making it harder to treat infections. At the same time, evolution of antibiotic resistance is probably the best-documented case of contemporary evolution. To date, research on antibiotic resistance has largely ignored the complexity of interactions that bacteria engage in. However, in natural populations, bacteria interact with other species; for example, competition and grazing are import interactions influencing bacterial population dynamics. Furthermore, antibiotic leakage to natural environments can radically alter bacterial communities. Overall, we argue that eco-evolutionary feedback loops in microbial communities can be modified by residual antibiotics and evolution of antibiotic resistance. The aim of this review is to connect some of the well-established key concepts in evolutionary biology and recent advances in the study of eco-evolutionary dynamics to research on antibiotic resistance. We also identify some key knowledge gaps related to eco-evolutionary dynamics of antibiotic resistance, and review some of the recent technical advantages in molecular microbiology that offer new opportunities for tackling these questions. Finally, we argue that using the full potential of evolutionary theory and active communication across the different fields is needed for solving this global crisis more efficiently.

This article is part of the themed issue ‘Human influences on evolution, and the ecological and societal consequences'.

## 1. Introduction

Human medicine and food production are heavily reliant on the effective use of antibiotics. The rise of antimicrobial resistance in human and animal pathogens poses a serious threat to human health and food production, respectively, as traditionally employed antibiotics are becoming ineffective in the face of rapidly evolving bacteria populations [1]. The increased problem of antimicrobial-resistant bacteria is linked to the introduction of antibiotics to clinical or farming use, and treating human/animal pathogens with antibiotics is expected to directly affect the frequency of resistance to those antibiotics in these pathogens [2–5]. Indeed, the evolution of antibiotic resistance is probably the best-documented case of contemporary evolution in action (see also [6] for other examples). Antibiotic resistance is currently considered a major threat to human health globally [7], but very little is known about what are the ecological and evolutionary consequences of human use of antibiotics in the wild. This is non-trivial given that spillover of antibiotic use to natural and semi-natural environments may have profound implications on the distribution of antibiotic resistance genes in natural populations. As such, natural bacteria populations may serve as environmental reservoirs of resistance determinants, but how resistance evolves, and how resistance genes are maintained and dispersed in the wild is poorly understood. Unravelling these mechanisms will be critical for predicting emerging resistant pathogens as we can expect the existence of continuous feedback loops from clinical and farming environments to nature and back [8]. Understanding the drivers of these dynamics may prove critical for preventing and treating the antibiotic resistance problem.

Bacteria are prevalent across all habitats, with the total number of bacterial species estimated to exceed one million [9]. However, only some 10–20 species of bacteria are currently known to be specialist human pathogens, while several hundred bacteria species are considered opportunistic pathogens that may cause human disease in certain conditions [10]. Many of the bacteria associated with humans (pathogenic, mutualists or commensals) also have other hosts, including a wide variety of livestock and wildlife species, or they can be found in the wider environment (sapronotic) or both. *Escherichia coli* is an obvious example of such a species [10,11]. Solving the problem of antimicrobial resistance in a single environment, such as in the clinic, will prove ineffective given that in bacteria, mobile genetic elements (MGEs) and the drugs themselves move among human, animal and environmental compartments [12]. Although anthropogenic activity has been shown to increase the antibiotic resistance gene abundance in the environment [13], even in the pre-antibiotic era bacteria in natural habitats have harboured antibiotic resistance genes independently of human activities [14–17]. Hence, the determinants of antibiotic resistance exist naturally [18], and an understanding of the eco-evolutionary dynamics of antibiotic resistance in natural communities is required for tackling the problem from a human health and food production perspective.

**Figure 1.**
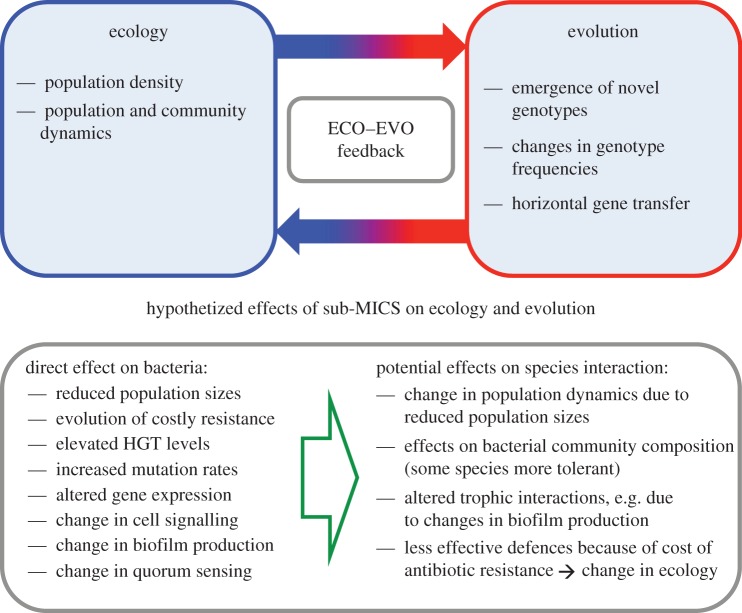
Schematic of eco-evolutionary feedback loop and role of sub-MIC (minimal inhibiting concentration) antibiotics directly on bacteria and indirectly on trophic interactions. (Online version in colour.)

**Figure 2.**
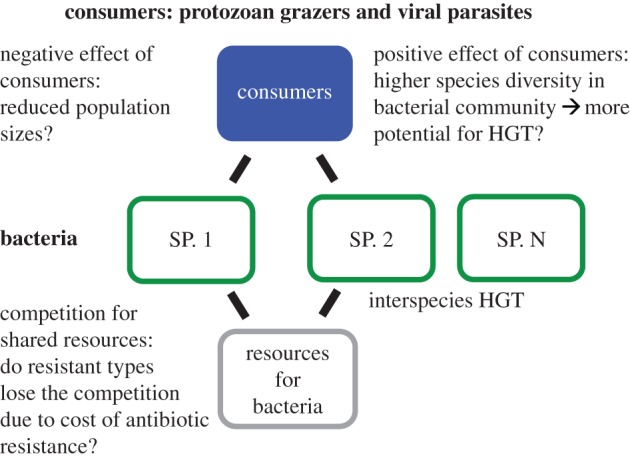
A presentation of hypothetical scenarios of how species interactions may alter the spread of antibiotic resistance in microbial communities. (Online version in colour.)

The emergence and spread of antimicrobial resistance has become an active area of research in recent years [19]. Considerable advances have been made in understanding the mechanisms by which variable levels of antibiotics influence bacteria, and the mechanisms by which antibiotic resistance evolves and spreads in bacterial populations (as reviewed in [20,21], respectively). The key aim of this review is to connect some of the well-established key concepts in evolutionary biology and recent advances in the study of eco-evolutionary dynamics to research on antibiotic resistance (figure 1). We use this framework to highlight how important the community context—e.g. competition and grazing—that bacteria in natural populations are embedded in, may be for how antibiotic resistance evolves and spreads (figure 2). We further discuss how antibiotic leakage to natural environments can radically alter bacterial communities, thereby potentially altering eco-evolutionary feedback loops in microbial communities. We will identify major gaps in our knowledge regarding these processes and pinpoint future perspectives so that we can move towards a predictive framework in the eco-evolutionary study of antibiotic resistance. We will also briefly describe the recent major advances in technology that are bringing us closer to this aim.

## 2. An eco-evolutionary perspective of antibiotic resistance: key concepts and terminology

Antibiotic resistance is a global health crisis with multiple dimensions. Generating effective solutions to this crisis requires active collaboration and communication among scientists representing different disciplines. Unifying the language that we use and having a clearly defined terminology among physicians, microbiologists, evolutionary biologists and environmental scientists—among others—is a necessity so that we can proceed in solving the problem of antibiotic resistance in the most efficient manner. Below we describe some of the key terminology we use, and advocate their use as defined here across disciplines.

### (a) Using the term evolution in the context of antibiotic resistance

Although the increase in resistance of human pathogens to antimicrobial agents is one of the best-documented examples of contemporary evolution in action, the actual word ‘evolution’ is rarely used in the papers describing this research [22]. As demonstrated by Antonovics *et al*. [22], the word ‘evolution’ is used with different frequency by evolutionary biologists versus researchers in the medical fields. More often antimicrobial resistance is described to ‘emerge’, ‘arise’ or ‘spread’ rather than ‘evolve’. The ambiguous use of terminology creates points of confusion that may simply reflect differences in traditions among fields. However, it may be that ‘evolution’ is considered to be a rather non-specific term meaning ‘gradual change,’ and that ‘emergence’ more precisely incorporates the key components of the evolutionary process, namely, mutation, recombination and/or horizontal transfer of resistance. It is also possible that the failure to use the word ‘evolution’ reflects the flawed idea that evolutionary processes are long past, slow and undetectable. This is worrying as it may have a direct impact on how the public perceives the importance of evolutionary processes in our everyday lives [22]. To avoid further confusion and to promote concise dialogue both within the scientific community, and between researchers and the general public, we argue that researchers across disciplines should adopt the term ‘evolution’ when describing the evolution of antibiotic resistance in bacteria.

### (b) Eco-evolutionary dynamics

The potential for eco-evolutionary feedback loops in determining the interaction between ecological and evolutionary dynamics has been increasingly recognized in recent years [23–27]. While the potential of species to adapt to ecological conditions has long been realized, the effect of rapid evolutionary change on ecological dynamics is still poorly understood. In part, this is due to the fact that traditionally evolution has been viewed as a slow process operating at a timescale that is very different from ecological time [28]. From such a perspective, ecological dynamics would play out as if evolution was not occurring, as evolutionary change would be non-significant on the ecological time-scale. Likewise, short-term fluctuations in ecological variables would average out over evolutionary timescales, and only the long-term average would affect evolution [29]. However, it is becoming increasingly clear that evolutionary change can be extremely rapid, in the case of horizontal gene transfer (HGT) practically instantaneous, and there are compelling examples of this in a diversity of traits ranging from life histories to behaviour and physiology [30]. Moreover, a rapidly increasing number of studies suggest that eco-evolutionary dynamics and feedbacks have the potential to play a prominent role in the dynamics of populations and in species interactions [25,31]. Hence, as we discuss below, adapting an eco-evolutionary framework for the study of antibiotic resistance offers tremendous potential for increasing our understanding of the rapid evolution we have witnessed in bacterial populations in antibiotic resistance.

### (c) Horizontal gene transfer is a key mechanism for the evolution of antibiotic resistance

HGT is the process where bacterial cells can exchange genetic material. Antibiotic resistance genes are often carried on MGEs (plasmids, transposons or integrons) that act as vectors transferring genetic information between bacteria and even between species boundaries. Three main mechanisms for HGT are conjugation, transformation and transduction. In *conjugation*, DNA is transferred in cell contact. *Transformation* does not require cell-to-cell contact but short fragments of naked DNA are taken up by naturally transformable bacteria. *Transduction* involves transfer of DNA via bacteriophages. Essentially, these mechanisms allow extremely rapid evolution since with HGT bacteria can change their genetic make-up practically instantly regarding a trait that has dramatic effect on fitness in the presence of antibiotics. While HGT is the main mechanism by which bacteria acquire antibiotic resistance, another key mechanism is chromosomal mutations. The main difference between these mechanisms is how fast resistance adaptation occurs in bacteria.

### (d) Sub-inhibitory antibiotic concentrations

Most often the aim of antibiotic usage is to kill the bacteria by using high enough concentrations of the given antibiotic. The key concept here is the minimal inhibiting concentration (MIC), which is the concentration that inhibits visible bacterial growth [32]. However, massive use of antibiotics has created antibiotic gradients where lower than inhibiting concentrations can also be observed in natural habitats. Even though under these concentrations the bacterial population does not go extinct, they can have important effects on bacteria (see [20] for a comprehensive review on the topic). These sub-MICs have been suggested, for example, to select for resistance, increase the bacterial mutation rates, increase phenotypic and genotypic variability and affect biofilm formation, to name but a few. In addition, importantly, sub-MICs promote the maintenance of horizontally transmitted resistance genes [20]. Finally, these sub-MICs can be especially important in the multispecies communities where even small changes in species interaction can have cascading community-level effects.

### (e) Anthropogenic antibiotic use creates selection for resistance in the environment

The correlation of therapeutic antibiotic use and antibiotic resistance level in human populations is well documented [33,34]. Although in both humans and animals, the first target of selection on antibiotic resistance is inside the body, anthropogenic activity results in elevated concentrations of antibiotics in the environment as well because (i) most of the antibiotic compound used is excreted from the body [35]; (ii) the animal feed containing the antibiotic is not all eaten; and (iii) there is considerable spillover from the antibiotic production industry [36]. Excreted antibiotics can end up in wastewater treatment plants which are capable of degrading the compound only partially [37]. At the same time, wastewater treatment plants are possible hotspots of HGT between bacterial species since bacteria coming from different environments are in close contact [38]. Antibiotics from aquaculture end up directly in the aqueous environment either from uneaten feed or via excretion. Antibiotics used in livestock end up in the environment from the manure. Anthropogenic activity clearly results in the situation where antibiotic compounds are present practically everywhere in varying concentrations and in some compartments of the environment antibiotic concentrations can even reach therapeutic levels [36]. However, even sub-MICs can select for antibiotic resistance [20], and hence the effect of large-scale antibiotic use has created an environment where selection pressure for antibiotic resistance is widespread. Moreover, antibiotic resistance genes spreading by HGT can be selected upon not only by antibiotics but also by, for example, metals or biocides since one mobile genetic element usually contains multiple resistance genes [21]. Indeed, the co-selection or co-selection potential of antibiotic resistance genes with metals [39] and biocides [40] has been verified.

The problem of human-mediated selection for antibiotic resistance in the environment can go well beyond the threats to human health and food production. Antibiotic use shapes the microbial community associated not only with humans and human-linked animals but in the environment by enrichment of antibiotic-resistant bacteria at the expense of antibiotic-sensitive bacteria. The possible effects of this large-scale event for the processes driven by microbes are yet to be seen.

## 3. Knowledge gaps and future directions

In this section, we outline how an understanding of eco-evolutionary dynamics could help in tackling the problem of antibiotic resistance and what are the key gaps in knowledge related to this.

### (a) Evolutionary response to antibiotic selection in natural environments

The essence of the antibiotic resistance problem is that bacterial populations react to selection pressures caused by antibiotics by evolving resistance against antibiotics. This phenomenon is well documented in the case of high, therapeutic antibiotic concentrations, and several studies have also demonstrated how resistance evolves in the presence of sub-MICs [41,42]. The evolutionary responses are expected to differ, depending on whether bacteria are exposed to high (more than MIC) or low (less than MIC) antibiotic concentrations. Under high antibiotic concentrations, the antibiotic resistance gene must already be present in the population or the evolution of resistance must be very rapid so that the population can survive via changes in genotype frequencies (susceptible die and resistant take over). Hitting hard with high antibiotic concentrations has been the commonly used practice when treating infections. However, from the point of view of evolutionary theory, the use of extreme force generates a selection scenario that may be driving the most feared outcome, i.e. the emergence of those pathogens that are not responsive to existing drugs. Indeed, recently Day & Read [43] have argued that nothing in evolutionary theory supports this as a good rule of thumb. Instead, by taking a modelling approach, they show that the only generality is to either use the highest tolerable drug dose or the lowest clinically effective dose; that is, one of the two extremes of the therapeutic window. In the case of selection under sub-MIC, bacteria have time to evolve de novo mutations or acquire resistance by other means (HGT). Under sub-MICs, the key factor determining if the resistant type increases in frequency is the cost of resistance [44]. It has been demonstrated that positive selection can occur under extremely low antibiotic concentrations. For example, it has been experimentally demonstrated that sub-MICs that are at the scale of a few per cent of the MIC can favour resistant genotypes [45].

Overall, in natural environments sub-MIC antibiotics are one probable reason for enrichment of antibiotic-resistant bacteria even when the concentrations are extremely low. In addition, in the environment, there may be sub-MICs of different antibiotics which can cause synergistic selection pressures. However, to date, the empirical evidence for how MICs of antibiotics act as a selective agent in natural bacterial communities is still restricted. Obtaining reliable estimates can be challenging, since, for example, antibiotic concentrations in soil can be very patchily distributed and bulk estimates do not provide a comprehensive picture. We, however, argue that there is a pressing need for studies that combine sampling (while taking the spatial scale into account) and surveys of antibiotic concentrations and resistance genes in natural environments with controlled experiments using realistic levels of antibiotics to mimic selection pressures that bacterial communities are faced with in nature.

### (b) Antibiotic-resistant bacteria are part of ecological communities

In natural settings, bacteria harbouring antibiotic resistance genes are part of complex communities where they interact with other species. However, at the moment, we are largely missing a population-level perspective on what processes constrain and drive antibiotic resistance in species-rich communities when complex species interactions are present. For example, in the case of spread of antibiotic resistance genes via HGT, microbial communities can be seen as gene-sharing networks where species composition and diversity can be a major factor determining how resistance genes spread [46]. In the case of species diversity, some recent studies suggest that certain species are pivotal hub-species, which promote the spread of antibiotic resistance genes, indicating that species composition can be critical [47,48]. Understanding these factors can be vital since a key unanswered question concerning the antibiotic resistance problem is how resistance genes are maintained in natural populations even though they are proposed to be costly to carry [49].

In addition to bacteria interacting with other bacteria, key interactions in microbial communities are those between bacteria and their natural enemies such as protozoan predators and the interactions between bacteria and their viral parasites (bacteriophages). For example, in natural microbial communities, protozoans form the most abundant and diverse group of predators feeding on bacteria. The most pronounced effect of protozoan predators and phage parasites is the mortality that they cause in bacterial populations. There is some experimental evidence demonstrating that protozoan predation, phage parasitism and maintenance of antibiotic resistance genes can be linked. One study shows that the plasmid was better maintained when the protist predator was present [50], while another experimental study shows that the presence of phage limits the existence conditions of the conjugative plasmid [51]. Although the empirical evidence is currently limited, these findings highlight the need for understanding the potentially critical links between species interactions and HGT. Overall, one critical knowledge gap is that a community perspective that accounts for species interactions might be needed to better understand the spread of resistance genes between species and ultimately the whole antibiotic resistance problem. For some time, it has been known that ecological interactions can be altered by rapid evolution (e.g. [24]). Rapid evolution in species interactions readily happens in bacterial populations, facilitated by large population sizes and rapid generation times (e.g. [52–54]). In addition, ecological and evolutionary processes are occurring at the same timescale and several empirical examples have demonstrated eco-evolutionary feedbacks in bacteria communities, e.g. with protozoan and phage consumers (e.g. [55–57]). While these simultaneous ecological and evolutionary processes take place, bacteria might also evolve antibiotic resistance or carry costly resistance genes. There is evidence showing that plasmid carriage can hinder phage resistance evolution in bacteria [51]. Furthermore, Andersson & Hughes [49] propose that the cost of resistance, when antibiotics are not present anymore, might be so small that resistant genotypes are outcompeted very slowly since the fitness difference between resistant and susceptible types is very small. Furthermore, the sometimes uncontrolled and extensive use of antibiotics has resulted in sub-MIC antibiotic concentrations. It is feasible to assume that these environmental concentrations of antibiotics can alter the functioning of the microbial communities, both on ecological and evolutionary levels [58]. Any direct evidence, or even theoretical or conceptual work, on connections between sub-MICs and eco-evolutionary feedbacks is still missing. This information would, however, most probably be critical in understanding how antibiotic resistance spreads in natural bacterial communities.

### (c) Molecular analysis tools enable studying eco-evolutionary dynamics of antibiotic resistance in the wild

Development of molecular techniques has been crucial for increasing our understanding of the evolution of antibiotic resistance in microbial communities. Importantly, they are not subject to the culture bias caused by the fact that only a small minority of bacterial species can be cultivated with the current methodology [59]. Metagenomic analysis based on high-throughput DNA sequencing is essential for understanding the eco-evolutionary dynamics of antibiotic resistance in a community context [60]. Long-read sequencing technologies such as PacBio RS [61] and Oxford Nanopore MinION [62] are important as they facilitate the assembly of long contigs thereby facilitating identifying genomic areas under selection. Quantitative PCR is also a useful tool for quantifying antibiotic resistance genes. Array-qPCR is an especially efficient method for estimating the presence and amount of antibiotic resistance genes in different environments [63,64]. Sequencing of genomes of single cells [65] enables a detailed genetic analysis of the microbial cell without culturing and is therefore a vital method for studying evolution of antibiotic resistance in natural communities. With the recently described epicPCR method [66], it is now possible to connect a gene with its host at a single-cell resolution for gene–host analysis making it a very useful tool in studying the genetics of antibiotic resistance dynamics. Overall, single-cell techniques can become extremely useful if they are developed in the direction where several traits can be simultaneously monitored, potentially providing information about community composition and trait evolution (e.g. in species interaction in addition to antibiotic resistance) in a high-throughput manner.

## 4. Conclusion

A large amount of research has been conducted to better understand the emergence of antibiotic resistance, which is recognized as a serious and global problem. However, most of the research has not taken species interactions into account although in natural populations bacteria are embedded in species communities characterized by diverse interactions. Also, antibiotic leakage to natural environments has the potential to radically alter resistance evolution and microbial community dynamics and structure. With this review, we want to (i) highlight the potential importance of species interactions in the evolution of antibiotic resistance; (ii) discuss how low environmental antibiotic concentrations could affect the evolution of antibiotic resistance and indirectly trophic interactions; and (iii) unify some of the key terminology between evolutionary biologists and researchers in biomedical fields working on antibiotic resistance problem. Establishing direct links between the fundamental axes of eco-evolutionary dynamics and species interactions offers an exciting future venue of research and is needed to tackle the problem of antibiotic resistance.
